# Is Sentinel Lymph Node Biopsy a Viable Alternative to Axillary Lymph Node Dissection in Breast Carcinoma Patients Who Have Received Neo-Adjuvant Chemotherapy?

**DOI:** 10.7759/cureus.52698

**Published:** 2024-01-21

**Authors:** Rehena Sulthana, Akshita Singh

**Affiliations:** 1 Surgery, Jawaharlal Institute of Postgraduate Medical Education and Research, Puducherry, IND; 2 General Surgery and Breast Oncology, Narayana Health City, Bangalore, IND

**Keywords:** neoadjuvant chemotherapy (nact), nact, early breast carcinoma, axillary lymphnode dissection, sentinel lymphnode biopsy

## Abstract

Background

Sentinel lymph node biopsy (SLNB) is based on the hypothesis that lymph from a primary solid neoplasm drains into one or more sentinel nodes, which are the first nodes at risk for harbouring occult metastatic disease. Sentinel lymph node biopsy has replaced axillary lymph node dissection (ALND) as the standard method for axillary staging in clinically node-negative patients. It avoids the complications associated with ALND and allows assessment of nodal status in patients with clinically node-negative breast cancer.

Aims and objectives

The aim of this study is to determine the false negative rate and identification rate of SLNB in breast cancer patients who received neoadjuvant chemotherapy (NACT).

Materials and methods

We conducted a hospital-based prospective study that included 19 patients who presented with early breast cancer and were node-positive. Post NACT, intraoperatively, methylene blue and radiocolloid dye were injected in the subareolar region. During the surgery, the blue and hot nodes identified were dissected, sent for frozen section analysis, and subsequently submitted for histopathological evaluation. This was followed by a standard-level I/II/III axillary clearance with histopathological examination.

Results

The false-negative rate of SLNB is 25%. Sentinel lymph node biopsy was more accurate with stage II than stage III tumours, and in patients who downstaged from stage II to any stage following NACT, it was more accurate than downstaging from stage III.

The average number of sentinel nodes identified was 1.9, with the maximum being seven and the minimum being one. A total of 25 sentinel lymph nodes were identified in 13 patients, with an identification rate of 68.42%.

Conclusions

The main clinicopathological factors that influence the false negative rate of SLNB after NACT are axillary lymph node status, stage of the tumour at presentation, and tumour downstaging. For patients for whom sentinel nodes cannot be harvested, ALND should be done.

## Introduction

Breast cancer is a global health problem, with over one million cases being diagnosed every year. It is the second-leading cause of cancer-related deaths [1], accounting for about 1.4 million cases each year, next to lung cancer with approximately 40,000 deaths annually. However, with the advent of breast cancer screening, smaller lesions are being detected early and low-grade tumours are being treated early.

The clinicopathological features that determine the prognosis of breast carcinoma patients include age, menopausal status, tumour size, lymph node status, grade of tumour, lymphovascular emboli, extracapsular invasion, and molecular subtypes, with a strong association with disease-free and overall survival [2]. The number of lymph nodes positive for metastasis is directly proportional to tumour size. The incidence of nodal metastasis in patients with tumours less than 2 cm was up to 30%, and in those with tumours larger than 5 cm, it was up to 52% [3].

The presence of micrometastasis and isolated tumour cells has an important role in the prognosis of the disease. The presence of micrometastasis without any systemic therapy has a negative prognostic effect on the outcome, as shown by the Minimally Invasive Robotic Surgery, Role in Optimal Debulking Ovarian Cancer, Recovery, and Survival (MIRRORS) study [4]. Extracapsular extension of metastatic tumours in regional lymph nodes is associated with increased lymphovascular invasion, involvement of a larger number of lymph nodes with a negative impact on the clinical outcome, and higher local, regional, and distant recurrences [5].

The technique of sentinel lymph node biopsy (SLNB) was developed to overcome the morbidities associated with axillary clearance while still providing accurate staging information. The sentinel lymph node is the first node that drains the tumour. It is the most likely node to contain the metastatic disease, if present [6]. In the SNLB procedure, a radiocolloid dye, methylene blue dye, or both are injected in the circumareolar or peritumoral region. The sentinel node is identified as a blue node or a radioactive hot node with a gamma probe. If the histopathology of the sentinel node is negative for metastasis, then the chances that other nodes are involved are <1% [7], and hence axillary lymph node dissection can be avoided.

Axillary recurrence rates have been shown to be extremely low after a negative SLNB without axillary dissection, provided the false negative rate of the institute is less than 10% [7]. In breast cancer, the primary aim of SNLB is to reduce the morbidity associated with routine axillary dissection. Sentinel lymph node biopsy replaces routine axillary dissection for breast cancer and identifies patients who may benefit from a therapeutic lymph node dissection [8, 9]. Axillary lymph node status is a very important prognostic factor in breast cancer patients. For this reason, axillary lymph node dissection (ALND) has been an integral component in the surgical treatment of invasive breast cancer. Not only does ALND help with locoregional control of the disease, but it also helps in staging the patient accurately, thus helping in assessing the need for adjuvant radiotherapy and systemic treatment. However, ALND results in significant morbidity, such as lymphedema, seroma formation, arm weakness, decreased shoulder range of motion, and neurological changes [10]. However, axillary lymph node clearance remains the standard of care for patients with locally advanced breast cancer, inflammatory breast carcinoma (T4d disease), patients with a positive sentinel lymph node (T3 tumours, ≥3 positive sentinel nodes), and patients scheduled for a mastectomy [11].

Neoadjuvant chemotherapy (NACT), in the form of systemic chemotherapy or hormonal therapy before surgery, results in a significant reduction in tumour size in patients. Neoadjuvant chemotherapy downstages tumours, in turn increasing the chances of breast conservation surgery. Neoadjuvant chemotherapy is used in cases where the patient wishes for breast conservation but is not eligible for the same due to large tumours or an inappropriate breast-to-tumour ratio. Various studies show that the presence or absence of residual disease in the breast and axilla is a strong predictor of overall survival in patients. Neoadjuvant chemotherapy also helps to eradicate the micrometastasis in regional lymph nodes in about 40% of patients.

## Materials and methods

Study site

The study was conducted at the department of oncoplastic breast surgery, Mazumdar Shaw Medical Centre, Narayana Health City, Bangalore, India.

Study population

Patients admitted to the department of general surgery and oncoplastic breast surgery in Narayana Hrudayalaya Hospital, Bangalore, who presented with breast cancer, received NACT, and were clinically and radiologically node-negative post chemotherapy were included.

Study design

This was a hospital-based prospective study conducted in the department of oncoplastic breast surgery at Mazumdar Shaw Medical Centre, Narayana Health City, Bangalore.

Study duration

The study spanned eight months (November 2018 to June 2019).

Inclusion criteria

Patients over the age of 18 undergoing elective breast surgery post NACT with pathologically (fine needle aspiration cytology (FNAC)/core needle biopsy) proven breast cancer without any clinically palpable nodes, at any stage, were included in the study.

Exclusion criteria

Patients below the age of 18 years, pregnant or lactating patients, patients with previous breast surgery that interfered with lymphatic drainage, patients who were known to be allergic to methylene blue dye, and patients who did not consent to the procedure or surgery were excluded from the study.

Study methods

This study included women over 18 years of age who attended the outpatient department of oncoplastic breast surgery at Mazumdar Shaw Medical Centre, Narayana Health City, Bangalore. An institutional ethical committee meeting was held on October 31, 2018, by the Narayana Health Academic Ethics Committee, and approval was obtained for conducting this study (approval number: NHH/AEC-CL-2018-313). Women with breast cancer who received NACT and were subsequently clinically and radiologically node-negative were selected for the study. Inclusion and exclusion criteria were applied to all patients. These patients were subjected to the required preoperative investigations.

In this study, two types of dye (methylene blue and radiocolloid dye) were injected into the subareolar region. Intraoperatively, 2 mL of methylene blue was injected into the subareolar region, followed by five minutes of gentle massage. During the surgery, the axillary nodes that were stained with blue dye were dissected, labelled as blue nodes, and sent for frozen section analysis. Around 0.5 ml (1 millicurie) of radiocolloid material was injected into the subareolar region preoperatively. A gamma probe was used to detect nodes with radiocolloid uptake (>10% of the count at the primary injection site), labelled as hot nodes, and also sent for frozen section analysis. Subsequently, a standard-level I/II/III axillary dissection was performed. All specimens were labelled accordingly and sent for histopathological examination.

Statistical methods

The sample size was calculated using N Master software version 2.0 (Informer Technologies Inc., Los Angeles, CA). Based on the expected false negative rate of 5.9% from the previous study by Hunt et al. [12], precision was measured at 10% with a confidence interval of 95%. The estimated sample size was 19.

Statistical analysis

Data were entered into a Microsoft Excel (Microsoft Corp., Redmond, WA) data sheet and analysed using IBM SPSS software version 22 (IBM Corp., Armonk, NY). Categorical data were represented in the form of frequencies and proportions. The chi-square test was used as a test of significance for qualitative data. Continuous data were represented as the mean and standard deviation. A p-value (probability that the result is true) of <0.05 was considered statistically significant after assuming all the rules of statistical tests.

Statistical software (Microsoft Excel, IBM SPSS version 22) was used to analyse the data. EPI Info (CDC, Atlanta, GA), OpenEpi (OpenEpi: Open Source Epidemiologic Statistics for Public Health, Version. www.OpenEpi.com, updated 2013/04/06), MedCalc (MedCalc Software Ltd., Ostend, Belgium), and Mendeley Desktop (Elsevier, London, UK) were used to estimate sample size and reference management in the study.

## Results

Of the 19 patients included in the study, the most common age group affected by breast cancer was 40-50 years of age (31.57%). The mean age at presentation was 48.1 years. Table 1 provides the distribution of the disease among different age groups in this study.

**Table 1:** Distribution of patients according to their age n: number; %: percentage

Age in years	No. of patients (n)	Percentage (%)
<40	5	26.31
40-50	6	31.57
51-60	4	21.05
>60	4	21.05
Total	19	100.0

Among the 19 female patients, post-menopausal women were more common, with 73.68% (n=14). Premenopausal women were about 26% (n=5). The most common symptom of breast cancer in our study was breast lumps (n=17, 89.47%), followed by breast pain (n=2, 10.52%). The incidence of right-sided breast complaints (n=11, 57.89%) appeared to be higher than that of left-sided breast complaints (n=7, 36.84%). Only one patient presented with symptoms in both breasts (5.26%). Most women visited our clinic between one month and one year after the onset of symptoms (73.68%; n=14). Women who visited the clinic within one month of symptoms accounted for only 10.52% (n=2). Around 15.78% of the patients visited the clinic after one year of the onset of symptoms (n=3). This clearly demonstrates a delay in seeking treatment after the onset of symptoms.

Of the 19 patients in our study, 73.68% belonged to the T2 group (n=14) and 26.31% belonged to the T3 group of tumours (n=5). No patients were identified during the early T stage of the disease, and 68% of them were clinically node-positive (n=13) and 32% of them were node-negative (n=6). Out of the 19 patients who received preoperative chemotherapy, four patients received the adriamycin, cyclophosphamide + docetaxel, cisplatin (AC+DC) regimen (22%), 12 patients received the adriamycin, cyclophosphamide + paclitaxel, carboplatin (AC+PC) regimen (63%), two patients received the Taxotere (docetaxel), carboplatin, Herceptin (trastuzumab) (TCH) regimen (10%), and one patient received the 5 fluorouracil, epirubicin, cyclophosphamide + paclitaxel, carboplatin (FEC+PC) regimen (5%). In our study, patients presented with grade II and grade III tumours, 68.4% (n=13) and 31.57% (n=6) respectively. There were no patients who presented with grade I tumours in our study.

Out of the 19 patients who underwent preoperative neoadjuvant chemotherapy, 31.5% (n=6) of the patients responded well to chemotherapy with no residual lesion; 26.3% of the patients presented with residual nodularity (n=5). Only six patients were found to be T2 (31.5%) after chemotherapy, as opposed to 14 patients before chemotherapy (73.68%). A T1 tumour was found in only one patient (5.26%), and in one patient (5.26%) the tumour was found to have increased in size after receiving chemotherapy. Eighteen patients experienced a decrease in tumour size after NACT. Following NACT, out of the 13 patients who were node-positive at the time of clinical presentation, only two of them continued to remain node-positive (15.38%), and the remaining 11 patients turned out to be node-negative clinically (84.61%). Those who were node-negative before NACT continued to be node-negative (n=6).

Sentinel lymph nodes were identified in 13 out of 19 patients, accounting for an identification rate of about 68.42%. In the remaining six patients, no sentinel nodes were identified. The average number of sentinel lymph nodes identified in this study was 1.9, with the maximum being seven nodes and the minimum being one. A total of 25 sentinel lymph nodes were identified in 13 patients, and no sentinel lymph nodes could be identified in about six patients. Tables 2-3 show the identification rate and the number of sentinel lymph nodes identified in the patients included in this study.

**Table 2:** Sentinel lymph node Identification

Type of node	Patients identified (out of 19)
Hot node	1
Blue node	4
Hot+blue node	11
Total sentinel nodes	13

**Table 3:** Identification of sentinel nodes by dual technique n: numbers; %: percentage

Number of nodes/patient (n)	Number of patients (n)	Percentage (%)
1 node	6	46.15
2-5 nodes	6	46.15
>/= 6 nodes	1	7.69
Total	13	100

Around 57.89% of the patients underwent breast-conserving surgery with axillary clearance (n=11), whereas the remaining 42.10% underwent modified radical mastectomy (n=8). The false-negative rate identified in this study is 25%. In the remaining six patients in whom no sentinel nodes were identified, four of them had a positive ALND (75%).

All the patients who belonged to stage II tumours at the time of presentation had a correlation between SLNB and axillary nodal status (n=5). Two out of eight patients who have stage III disease did not have an accurate sentinel lymph node histopathology examination (HPE) report. Table 4 shows the association between prechemotherapy clinical staging and SLNB-ALND correlation.

**Table 4:** Association between prechemotherapy clinical staging and correlation between SLNB and rest of axillary lymph node status SLNB: sentinel lymph node biopsy; n: number of patients *SLNB was identified only in 13 patients; p-value < 0.05

Prechemotherapy clinical stage		SLNB Correlation		Total (n)	p-value
		Yes (n)	No (n)		
	II (n)	5	0	5	0.37
	III (n)	6	2	8	

Around nine out of 11 patients who downstaged after receiving chemotherapy had a correlation between the SLNB and the rest of the axillary node HPE (81.8%). All patients who did not downstage had an accurate SLNB HPE report (n=2). Six out of eight patients who downstaged from stage III to any stage after receiving chemotherapy had an accurate SLNB HPE report (75%), whereas 100% of those who downstaged from stage II had an accurate SLNB report (n=3).

Out of the total 19 patients, the final size of the tumour in the HPE report was correlating with clinical examination in around nine patients (47%). In 10 of the patients, no correlation was found (53%). In around five patients (26.3%) who had tumour-positive status in HPE, a clinical lesion was not found. Out of the total 19 patients, the final size of the tumour in the HPE report was correlating with ultrasound examination only in seven patients (36.8%). In 12 of the patients, no correlation was found (63.15%). In around two patients (10.56%) who had tumour-positive status in HPE, no lesion was found in ultrasound. Out of the total 19 patients, the final size of the tumour in the HPE report correlated with mammography (MMG) tumour size in only six patients (31%). In 13 of the patients, no correlation was found (68.4%). Out of four patients who did not show any lesions in the mammogram, one patient turned out to be tumour-positive in the HPE report (25%). Out of 19 patients, axillary nodes were not palpable in about 17 patients after receiving NACT. Out of these 17 patients, nine of them had nodal metastasis in HPE (53%). Eight of them, who were node-negative clinically, stayed node-negative histopathologically (47%). Only eight patients were found to have a correlation between USG and HPE nodal status. In 11 patients out of 19 of them, axillary nodes were identified by ultrasound out of which five patients had metastatic disease in microscopic examination (45%), in the remaining eight patients in whom no nodes were identified in USG, 50% turned out to have metastatic deposits in lymph nodes in histopathological examination. Nine out of 19 patients (47%) were correctly identified as node-negative or positive by USG, which was confirmed by the HPE report. Table 5 demonstrates the correlation between USG and HPE in identifying the nodal status in post-chemotherapy patients.

**Table 5:** Association between HPE nodal status and USG nodal status (post chemotherapy) USG: ultrasonography; HPE: histopathological examination; n: number of patients p-value <0.05

HPE nodal status		USG nodes		Total (n)	p-value
		YES (n)	NO (n)		
	Positive (n)	5	4	9	1.0
	Negative (n)	6	4	10	

## Discussion

Of the 19 patients included in the study, the most common age group affected by breast cancer was between 40 and 50 years of age (32%). The mean age at presentation was 48.1 years. Toi et al. noted similar results: the peak incidence of breast cancer is at about 45-50 in the Asian population, whereas in Western countries the incidence continues to increase even at older ages [13]. Among the 19 female patients, post-menopausal women were more common (74%, n=14). Premenopausal women were about 26% (n=5) of the total participants. Other studies by Spitale et al. in Switzerland and Haque et al. in the Caucasian population also showed post-menopausal women to have more association with breast cancer, with 68.7% and 76.0%, respectively [14, 15]. Most women visited our clinic between one month and one year after the onset of symptoms (74%; n=14). Women who visited the clinic within one month of symptoms accounted for only 10% (n=2). Around 16% of the patients visited the clinic after one year of the onset of symptoms (n=3). This clearly demonstrates a delay in seeking treatment after the onset of symptoms.

The most common symptom of breast cancer in our study was breast lumps (n=17, 89.47%), followed by breast pain (n=2, 10.52%). The incidence of right-sided breast tumours (n=11, 57.59%) appeared to be higher than that of left-sided breast tumours (n=7; 36.54%). Only one patient presented with bilateral symptoms. This was in contrast to the study by Dane et al., in which the incidence was higher for left-sided breast cancer (57%) than right-sided breast cancer (43%) [16].

In our study, patients presented with grade II and grade III tumours, 68.4% (n=13) and 31.57% (n=6), respectively. There were no patients who presented with grade I tumours in our study. Studies like the study by Haque et al. in the Caucasian population showed grade II and grade III accounting for 63% and 24% of all tumours, respectively [15]. Huo et al. noted similar results in the African population, with 38% Grade II and 44% grade III tumours [17].

In our study, most patients belonged to stage III disease with 68.42% (n=13) followed by stage II disease with 31.57% (n=6). There were no patients with stage I/0 disease in our study, compared to the study by Huo et al. in the Chinese population, which showed stage IIa was more common among them with 33.85%, followed by stage I with 31.21%, then stage IIb with 19.61%, stage III and IV with 9.75%, and stage 0 with 2.26%. A study by Munjal et al. on the Indian population showed that 64% of cases belonged to stage III [18]. Of the 19 patients in our study, 73.68% belonged to the T2 group (n=14), and 26.31% belonged to the T3 group of tumours (n=5). No patients were identified during the early T stage of the disease. This is similar to the result of the study conducted by Kaur et al., where the T1 group was 8.8%, the T2 group was 67.6%, and the T3 tumour was 23.5% [19].

Out of the total 19 patients who presented, 68% of them were clinically node-positive (n=13), and 32% of them were node-negative (n=6). Similarly, 87.1% of patients were node-positive at the time of presentation in the study by Zhao et al., compared to 13% of node-negative patients [20]. Out of the 19 patients who received preoperative chemotherapy, four patients received the AC + DC regimen (22%), 12 patients received the AC + PC regimen (63%), two patients received the TCH regimen (10%), and one patient received the FEC + PC regimen (5%).

Out of the 19 patients who underwent preoperative neoadjuvant chemotherapy, 31.5% (n=6) responded well to chemotherapy with no residual lesion; 26.3% of patients presented with residual nodularity (n=5). Only six patients were found to be T2 (31.5%) after chemotherapy, as opposed to 14 patients prior to chemotherapy (73.68%). A T1 tumour was found in only one patient (5.26%) after chemotherapy, and in one patient (5.26%), the tumour was found to have increased in size after receiving chemotherapy. Eighteen patients had a decrease in tumour size following NACT.

Following NACT, out of the 13 patients who were node-positive at the time of clinical presentation, only two of them continued to remain node-positive (15.38%), and the remaining 11 patients turned out to be node-negative (84.61%). Those who were node-negative before NACT continued to be node-negative (n=6). In a study by Lee et al., 54.3% (44) of cases out of 81 patients turned node-negative after NACT, and 45.7% (37) of them remained node-positive [21]. Sentinel lymph nodes were identified in 13 out of 19 patients, accounting for an identification rate of about 68.42%. In the remaining six patients, no sentinel nodes were identified. A study conducted by Lee et al. also yielded a similar result, where the identification rate of SNB in patients who received preoperative NAC was 77.6% [21].

The average number of sentinel lymph nodes identified in this study is 1.9, with the maximum being seven nodes and the minimum being one. A total of 25 sentinel lymph nodes were identified in 13 patients, and no sentinel lymph nodes could be identified in about six patients. Among these six patients, four of them had a positive ALND. In a study conducted by Ban et al., an average of three nodes were identified, with the maximum being 14 and the minimum being one. They found that the accuracy of SNB increases with an increase in the number of SLNB (>4) [22]. Around 57.89% of the patients underwent breast-conserving surgery with axillary clearance (n=11), whereas the remaining 42.10% underwent modified radical mastectomy (n=8). This is in contrast to the study conducted by Ban et al., where 34.1% of patients underwent breast-conserving surgery and 65.9% of them underwent total mastectomy [22]. 

The false-negative rate of SLNB in our study was 25%. This is similar to the study conducted by Gimbergues et al. [23], where the false-negative rate of SLNB after NACT in N1-N2 patients was 29.6%. All patients who belonged to stage II tumours at the time of presentation had a correlation between SLNB and axillary nodal status (n=5). Two out of eight patients who had stage III disease did not have an accurate sentinel lymph node HPE report. A SLNB was identified in only 13 patients.

Nine out of 11 patients who downstaged after receiving chemotherapy had a correlation between the SLNB and the rest of the axillary node HPE (81.8%). Hundred percent of patients who did not downstage had an accurate SLNB HPE report (n=2). Six out of eight patients who downstaged from stage III to any stage after receiving chemotherapy had an accurate SLNB HPE report (75%), whereas 100% of those who downstaged from stage II had an accurate SNB report (n=3).

Out of the total 19 patients, the final size of the tumour in the HPE report was correlating with clinical examination in around nine patients (47%). In 10 of the patients, no correlation was found (53%). In around five patients (26.3%) who had tumour-positive status in HPE, a clinical lesion was not found. Zhi Fei Zhao et al. conducted a study in 440 patients where 15.9% of patients had no difference in tumour size between clinical examination and HPE. In contrast, around 84.1% of them found a difference in tumour size, with the minimum difference in size being 0.2 cm and the maximum being 3 cm [20].

Out of the total 19 patients, the final size of the tumour in the HPE report was correlating with ultrasound examination only in seven patients (36.8%). In 12 patients, no correlation was found (63.15%). In two patients (10.56%) who had tumour-positive status in HPE, no lesion was found on ultrasound. In another study conducted by Zhao et al. on the tumour size correlation between USG and HPE, no patients had an exact correlation in size. In about 22.73% of patients, tumour size was overestimated by USG, and in the rest, 77.2% of patients, tumour size was underestimated by USG [20].

Out of the total 19 patients, the final size of the tumour in the HPE report was correlated with MMG tumour size in only six patients (31%). In 13 of the patients, no correlation was found (68.4%). Out of four patients who did not show any lesions in the mammogram, one patient turned out to be tumour-positive in the HPE report (25%). In the Zhao et al. study, tumour size in MMG had an exact correlation in about 15.9% of patients, and an overestimation of tumour size was found in 36.36%. In around 47.72% of them, tumour size was underestimated by MMG. Hence, they concluded that MMG is a better modality than clinical examination and USG for detecting tumour size in breast cancer patients after NACT [20].

In the study conducted by Keune et al. [24], it was found that USG was more accurate (62/104, 59.6%) than MMG (33/104, 31.7%) in estimating the residual tumour. Out of 104 patients, USG was able to size tumours in 91.3% and MMG in 51.9%. Out of 19 patients, axillary nodes were not palpable in about 17 patients after receiving NACT. Out of these 17 patients, nine of them had nodal metastasis in HPE (53%). Eight of them, who were node-negative clinically, stayed node-negative histopathologically (47%). Only eight patients were found to have a correlation between USG and HPE nodal status. In a study conducted by Pamilo et al. [25] in 41 patients, the sensitivity of clinical examination was 32.3% in detecting positive nodes, as compared to 72.7% by USG and 38.9% by MMG.

In 11 patients out of 19, axillary nodes were identified by USG. Out of these, five patients had metastatic disease on microscopic examination (45%). In the remaining eight patients in whom no nodes were identified in USG, 50% of them turned out to have metastatic deposits in the lymph nodes in HPE. Nine out of 19 patients (47%) were correctly identified as node-negative or positive by USG, which was confirmed by the HPE report. In a study conducted by Kaur et al. [19], the USG evaluation of the axilla had a sensitivity of 100%, a specificity of 72.7%, a positive predictive value of 66.7%, and a negative predictive value of 100%. The diagnostic accuracy was 85%.

## Conclusions

The sentinel lymph node identification rate is better with the use of the dual technique. The SLNB was found to be accurate in nine out of 11 patients who downstaged following NACT and in two out of two patients who were in the same stage even after NACT. Sentinel lymph node biopsy is more accurate in patients who were converted from stage II to any stage following NACT compared to patients who were converted from stage III to any stage following NACT. The false-negative rate of SLNB in our study is 25%, and hence SLNB cannot be used as a reliable alternative to ALND.

Following NAC, the tumour size in HPE had a correlation of 47% with clinical examination, 36.8% with USG examination, and 31% with MMG. However, residual tumours could not be identified in about five patients by clinical examination and in two patients by USG examination. Out of the four patients who had no residual lesions in MMG, only one patient turned out to be tumour-positive in the HPE report. Hence, MMG is more reliable in identifying residual lesions following NACT. Only one out of 19 patients who did not have any residual disease in HPE was correctly identified by clinical examination, USG, and MMG. Eleven out of 19 patients had enlarged axillary nodes with features of metastasis in USG, out of which five patients were proven to have nodal metastasis by microscopic examination (45%). Four out of eight patients (50%) were wrongly identified as node-negative by USG but were found to have nodal disease in HPE.

The differing results could be a sampling bias as the sample size is small compared to other studies. Hence, the results of this cannot be extrapolated as this is single-center data in an urban south Indian population. The power of the study can be improved with increased numbers.

The main clinicopathological factors that influence the false negative rate of SLNB after NACT are axillary lymph node status, stage of the tumour at presentation, and tumour downstaging. For patients in whom sentinel nodes cannot be harvested, ALND should be done.
